# Drug-Resistant Malaria Parasites Introduced into Madagascar from Comoros Islands

**DOI:** 10.3201/eid1311.070235

**Published:** 2007-11

**Authors:** Didier Ménard, Armand Eugène Randrianarivo-Solofoniaina, Bedja Said Ahmed, Martial Jahevitra, Landy Valérie Andriantsoanirina, Justin Ranjalahy Rasolofomanana, Léon Paul Rabarijaona

**Affiliations:** * Institut Pasteur de Madagascar, Antananarivo, Madagascar; † Institut National de Santé Publique et Communautaire, Antananarivo, Madagascar; ‡ Direction Générale de la Santé, Moroni, Union des Comores

**Keywords:** malaria, Plasmodium falciparum, drug resistance, spread resistance, chloroquine, sulfadoxine-pyrimethamine, Indian Ocean, Comoros Islands, Madagascar, dispatch

## Abstract

To determine risk for drug-resistant malaria parasites entering Madagascar from Comoros Islands, we screened travelers. For the 141 *Plasmodium falciparum* isolates detected by real-time PCR, frequency of mutant alleles of genes associated with resistance to chloroquine and pyrimethamine was high. International-level antimalarial policy and a regional antimalarial forum are needed.

In the southwestern Indian Ocean, the epidemiologic features of malaria and antimalarial drug resistance differ considerably between islands that are very close geographically. Malaria remains a major public health problem in Madagascar and the Comoros Islands, whereas the situation is different on other nearby islands. Chloroquine resistance ranges from moderate in Madagascar (*1*–*3*) to high in the Comoros Islands (*4*,*5*), whereas pyrimethamine resistance is absent in Madagascar (*3*,*6*) but present at high levels in the Comoros Islands (*5*). The paradoxical situation of resistance to antimalarial drugs and prevalence of mutant-type parasites (for the *Plasmodium* *falciparum* chloroquine resistance transporter [*pfcrt*] gene, 90% of 76T alleles in the Comoros Islands vs. 3% in Madagascar; for the *P. falciparum* dihydrofolate reductase [*dhfr*] gene, 69% of 108N alleles in the Comoros Islands vs. 0% in Madagascar) (*2*,*5*,^7^) in such close geographic proximity led us to perform this study.

Historically, the Comoros Islands and Madagascar have been linked by human travel, and the importation of pathogens has already been documented, in the cholera epidemic of 1998–1999 (*8*) and in more recent outbreaks of arbovirus infection (*9**,**10*). Most travel between the Comoros Islands and Madagascar occurs through the seaport and airport of Mahajanga, the main city on the northwestern coast of Madagascar. To improve the monitoring of antimalarial drug resistance in Madagascar, we assessed the frequency of *P. falciparum* mutant alleles of genes associated with resistance to chloroquine (*pfcrt* and *P. falciparum* multidrug resistance 1 [*pfmdr-1*] gene) and pyrimethamine (*dhfr*) among travelers entering Madagascar from the Comoros Islands.

## The Study

**Figure 1:**
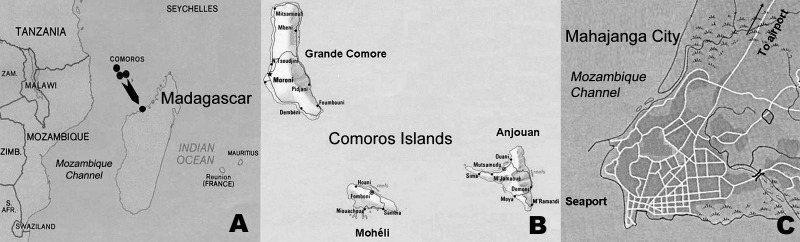
Regional map of the Indian Ocean, showing A) location of Comoros Islands and Madagascar; B) Comoros Islands; and C) location of Mahajanga seaport and airport, Madagascar.

The study was performed from March to July 2006, in the seaport and the airport of Mahajanga, on the northwest coast of Madagascar (Figure 1). These sites are the main communications crossroads between the Comoros Islands and Madagascar. The study was approved by the National Ethics Committee of the Ministry of Health and Family Planning of Madagascar.

All travelers from the Comoros Islands who consented to participate on arrival in Madagascar, regardless of their age, sex, nationality, and presence or absence of symptoms, were enrolled in the study. For each participant, a questionnaire was filled out and a finger-prick blood sample was collected. Rapid diagnostic tests (OptiMAL-IT, DiaMed AG, Cressier sur Morat, Switzerland) and thick/thin blood smears were performed in the field. Patients with a positive rapid test result were promptly treated with an artesunate and amodiaquine combination (Arsucam, Sanofi-Aventis, Paris, France), according to Madagascar’s national malaria policy.

Thick/thin blood smears were stained and analyzed by an experienced technician, without reference to rapid test results. A minimum of 200 consecutive fields were counted for each thick blood film before a slide was classified as negative. The number of parasites in thick blood films was determined per 200 or 500 leukocytes, assuming 8,000 leukocytes/μL of blood. Thin blood smears were also examined for other *Plasmodium* spp.

Parasite DNA was extracted from blood samples by using the phenol/chloroform method. *P. falciparum* carriers were detected by real-time PCR in a RotorGene 3000 thermocycler (Corbett Life Science, Sydney, New South Wales, Australia), as described by Mangold et al. (*11*). PCR and restriction fragment length polymorphism analyses were performed for 3 genes (codon 108 of *dhfr*, codon 76 of *pfcrt*, and codon 86 of *pfmd-1*) for the detection of mutant alleles. (Detailed descriptions of these methods are available from http://medschool.umaryland.edu/cvd/plowe.html.) Laboratory strains of *P. falciparum* were used as controls (positive and negative) and included in all PCR and enzyme digestion procedures (DNA from the W2, HB3, and 3D7 reference strains from the Malaria Research and Reference Reagent Resource Center, Division of Microbiology and Infectious Diseases, National Institute of Allergy and Infectious Disease, National Institutes of Health, Manassas, VA, USA). Statistical analyses were performed by using SPSS software (SPSS Inc., Chicago, USA). Odds ratios were calculated from logistic regression parameter estimates, and p values were determined with the significance level set at p<0.05.

Among the 1,130 travelers registered on arrival in Mahajanga, 947 agreed to participate in the study (crude participation rate 83.8%). The baseline characteristics of the enrolled travelers are given as a function of nationality (Comorian or Malagasy) in the Table. The frequency of *P. falciparum* carriers was 0.5% (5/947) according to rapid diagnostic test, 3.2% (30/947) according to microscopy, and 14.9% (141/947) according to real-time PCR.

**Figure 2:**
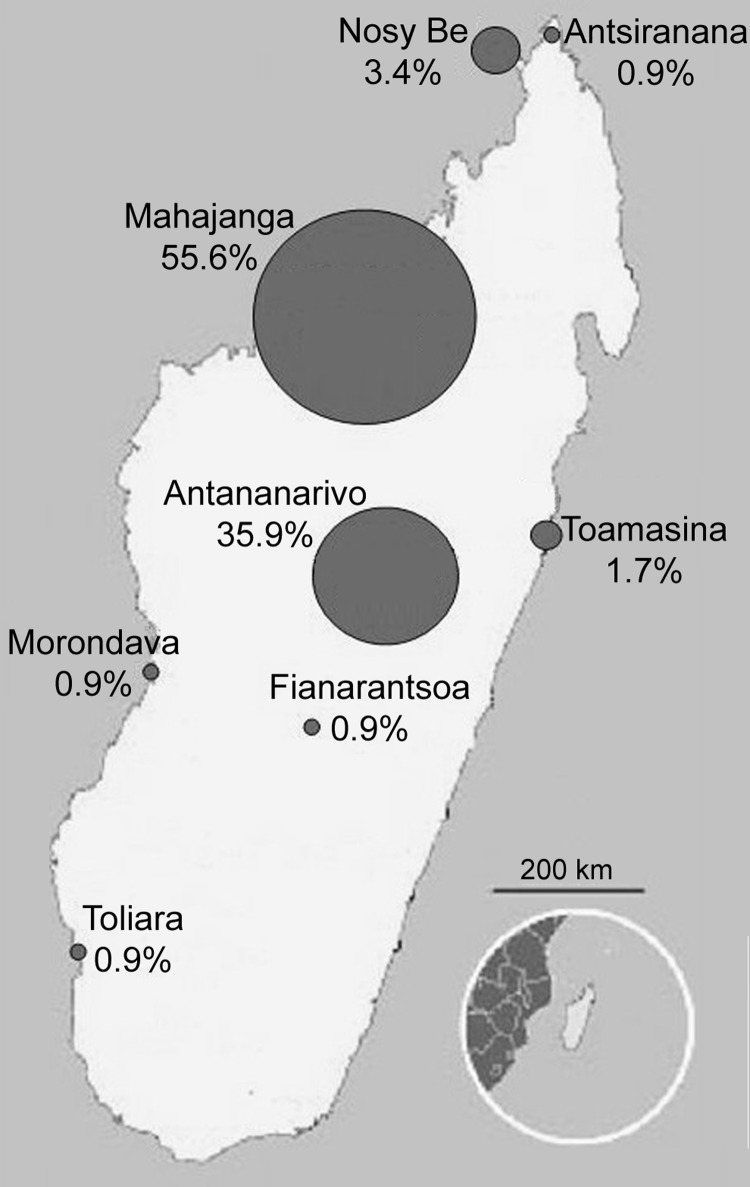
Map assessing the potential risk for spread of Plasmodium falciparum mutant-type alleles associated with resistance to chloroquine and pyrimethamine from the Comoros Islands to Madagascar, Mahajanga, Madagascar, 2006.

For the 141 *P. falciparum* isolates detected by real-time PCR, the frequency of the mutant alleles of genes associated with resistance to chloroquine (*pfcrt* and *pfmdr-1*) and pyrimethamine (*dhfr*) was 80.1% (113/141) for the 76T mutant allele of the *pfcrt* gene, 99.3% (140/141) for the 86Y mutant allele of the *pfmdr-1* gene, and 95.0% (134/141) for the 108N allele of the *dhfr* gene. More detail is provided in the Table. Univariate analysis of risk factors associated with the carriage of *P. falciparum* mutant alleles showed that for Comorian travelers, only a history of travel in Africa in the past 3 months was identified as significant (odds ratio 2.29, 95% confidence interval 1.27–4.13, p<0.01). We used these data to generate a map assessing the potential risk for the spread of *P. falciparum* mutant-type alleles in Madagascar (Figure 2).

**Table:** Baseline characteristics of enrolled travelers arriving in Mahajanga, Madagascar, from Comoros Islands, 2006*

Characteristic	Comorian travelers, n = 662	Malagasy travelers, n = 285	p value
Place of arrival, n (%)			
Airport	553 (83.5)	148 (51.9)	<10^–6^
Seaport	109 (16.5)	137 (48.1)	
Female	41%	29%	<10^–3^
Age			
Mean age in years (SD)	35.4 (14.7)	35.7 (12.5)	NS
<5 y, %	3.7	1.5	NS
Declared site of residence, %			
Grande Comore	65.2	NA	
Anjouan	31.2	NA	
Mohéli	3.6	NA	
Northwestern Madagascar	NA	64.1	
Central Highlands Madagascar	NA	23.8	
North Madagascar	NA	7.1	
East Madagascar	NA	3.6	
Southwestern Madagascar	NA	1.4	
Northeastern Madagascar	NA	0.4	
Mean duration of stay in days (SD)	63.5 (146.8)	111.8 (300)	NS
Place of stay in Madagascar, %			
Northwestern Madagascar	65.1	NA	
Central Highlands Madagascar	32.0	NA	
North Madagascar	1.9	NA	
East Madagascar	0.5	NA	
Southwestern Madagascar	0.5	NA	
Place of stay in Comoros Islands, %			
Grande Comore	NA	79.3	
Anjouan	NA	20.3	
Mohéli	NA	0.4	
Malaria symptoms at arrival, %†	3.3	3.9	NS
Medical history declared by travellers in the 3 previous months, %			
Suspected malaria	4.8	7.4	NS
Confirmed malaria	1.7	2.5	NS
Treated with antimalarial drugs	4.5	7.4	NS
History of travel in past 3 months			
In Africa, %	3.5%	4.9%	NS
In Asia, %	0.2%	3.9%	NS
No. malaria-positive samples (%)	105 (74.5)	36 (25.5)	
Frequency of mutant alleles,‡ %			
76T	82.0	75.0	NS
86Y	100.0	97.2	NS
108N	96.2	91.7	NS
Triple mutant type, **76T-86Y-108N**	82.2	71.4	NS
Double mutant type 1, 76K-**86Y-108N**	14.4	17.8	NS
Double mutant type 2, **76T-86Y**-108S	2.2	3.6	NS
Single mutant type, 76K-**86Y**-108S	1.1	3.6	NS
Wild type, 76T-86Y-108N	0	3.6	NS

*NS, not significant; NA, not applicable; **boldface** indicates mutant types. †Fever, headache, diarrhea, shivering, vomiting. ‡Associated with resistance to chloroquine (*pfcr*t and *pfmdr-1*) and pyrimethamine (*dhfr*).

## Conclusions

Despite some methodologic limitations (limited study period, limited number of passengers screened, taking into account only registered travelers), this study provides the first, to our knowledge, direct measurement of parasite movement between the Comoros Islands and Madagascar. This study thus enables an assessment of the potential threat of *P. falciparum* mutant allele parasites being introduced into Madagascar.

First, we noted that for detection of *P. falciparum* carriers, real-time PCR was 4.6 times more sensitive than microscopy and 30 times more sensitive than rapid diagnostic testing based on parasite lactate dehydrogenase detection, according to the threshold detection level of the techniques used (*11*,*12*). The results suggest that most of the *P. falciparum* carriers had low-level parasitemia. Second, most of the imported parasites carried resistance-associated mutations, consistent with the frequency of mutant forms of *P. falciparum* circulating in the Comoros Islands (*4*,*5*). Third, according to the places in which the Comorian travelers stayed and the places in which the Malagasy travelers lived, the potential area of antimalarial drug–resistant parasite spread was located in the northwestern area, which has a transmission season of >6 months per year (*13*).

On the basis of our findings, we suggest that antimalarial drug policy should be formulated at an international, rather than a national, level. Given that the Malagasy government’s goal is moving toward malaria elimination, international considerations in antimalarial policy would avoid the possibility of a coherent national policy being annulled by mutations originating in or spreading through neighboring countries. For example, the introduction of high-level chloroquine or pyrimethamine resistance from the Comoros Islands could compromise the use of the artesunate and amodiaquine combination as a first-line treatment for uncomplicated falciparum malaria or the use of sulfadoxine-pyrimethamine for intermittent preventive treatment in pregnant women and render these strategies useless.

In conclusion, we suggest the creation, as soon as possible, of a regional antimalarial forum in the Indian Ocean, similar to the East African Network for Monitoring Antimalarial Treatment (www.eanmat.org). Such a forum would enable the countries of the region to share national information on antimalarial drug efficacy, such as the prevalence of drug resistance molecular markers, and to debate proposed changes in national policy.
